# A Food-Derived Flavonoid Luteolin Protects against Angiotensin II-Induced Cardiac Remodeling

**DOI:** 10.1371/journal.pone.0137106

**Published:** 2015-09-01

**Authors:** Atsuko Nakayama, Hiroyuki Morita, Tomoko Nakao, Toshihiro Yamaguchi, Tomokazu Sumida, Yuichi Ikeda, Hidetoshi Kumagai, Yoshihiro Motozawa, Tsukasa Takahashi, Atsushi Imaizumi, Tadashi Hashimoto, Ryozo Nagai, Issei Komuro

**Affiliations:** 1 Department of Cardiovascular Medicine, Graduate School of Medicine, The University of Tokyo, Tokyo, Japan; 2 Department of Translational Research for Healthcare and Clinical Science, Graduate School of Medicine, The University of Tokyo, Tokyo, Japan; 3 Theravalues Corporation, Tokyo, Japan; Universidade Federal do Rio de Janeiro, BRAZIL

## Abstract

Oxidative stress has been implicated in cardiac remodeling (cardiac fibrosis and hypertrophy), which impairs cardiac function and metabolism; therefore, it is anticipated antioxidative compounds will have protective properties against cardiac remodeling. Luteolin (3’,4’,5,7-tetrahydroxyflavone), a widely distributed flavonoid found in many herbal extracts including celery, green pepper, perilla leaves and seeds, and chamomile, is a known to be a potent antioxidant and was previously demonstrated to exert an antifibrotic effect in the lungs and the liver. In this study, we clearly demonstrate that oral pretreatment with the higher-luteolin diet (0.035% (wt/wt)) protected against cardiac fibrosis and hypertrophy as well as a hyperoxidative state in Ang II-infused rats. In cardiac tissue, increased gene expression levels of TGFβ1, CTGF, Nox2, Nox4, ANP, and BNP induced by Ang II were restored by oral pretreatment of this high-luteolin diet. In cultured rat cardiac fibroblasts, H_2_O_2_-induced TGFβ1 expression and the phosphorylation of JNK were suppressed by luteolin pretreatment. In conclusion, food-derived luteolin has protective actions against Ang II-induced cardiac remodeling, which could be mediated through attenuation of oxidative stress.

## Introduction

Hypertensive cardiac remodeling, characterized by left ventricular hypertrophy and cardiac fibrosis, is a major risk factor for cardiovascular morbidity and mortality and a leading cause of chronic heart failure [1]. In particular, cardiac fibrosis, which is due to increased collagen formation by activated and differentiated cardiac fibroblasts (myofibroblasts) in response to various stresses, impairs contractile function, metabolism, and electrical coupling of myocardium, which profoundly contribute to heart failure and predispose the heart to arrhythmias and sudden cardiac death [2]. The prevention and/or treatment of excessive cardiac fibrosis could improve cardiac function and the prognosis [3–5].

Since the oxidative stress has been implicated in cardiac remodeling [6–10], antioxidative compounds are expected to have protective properties against cardiac remodeling. Among various food-derived antioxidative compounds, flavonoids are thought to be particularly valuable due to their multifunctional actions that include antioxidative, antiproliferative, and anti-inflammatory properties [11]. Luteolin (3’,4’,5,7-tetrahydroxyflavone), a widely distributed flavonoid found in many herbal extracts including celery, green pepper, perilla leaves and seeds, and chamomile is a known potent antioxidant and was clearly demonstrated to exert an antifibrotic effect in lung and liver [12,13]. Therefore, we have come to hypothesize that luteolin may be a potent candidate as a food-derived compound that can ameliorate pathological cardiac remodeling.

The aim of this study was to investigate the protective role of oral luteolin administration on angiotensin II (Ang II)-induced cardiac remodeling, focusing on the suppressive properties of luteolin against redox-sensitive pathways in cardiac fibroblasts.

## Materials and Methods

All animal experiments were approved by the University of Tokyo Ethics Committee for Animal Experiments and strictly adhered to the guidelines for animal experiments of the University of Tokyo.

### Angiotensin II-induced hypertension model

Male Sprague-Dawley rats (4 weeks of age; Saitama Experimental Animals Supply Co., Ltd., Saitama, Japan) were housed in a standard cage, with food and water available *ad libitum*, under standard laboratory conditions (lights on from 08:00 to 20:00). At 5 weeks of age, the rats were randomly divided into 4 groups as follows; control group, Ang II group, Ang II + low-dose luteolin group, and Ang II + high-dose luteolin group. For 3 weeks, the control group rats and the Ang II group rats were fed MF chow, and the Ang II + low-dose luteolin group rats and Ang II + high-dose luteolin group rats were fed MF chow supplemented with luteolin (Sigma-Aldrich Co. LLC, St. Louis, MO, USA) at concentrations of 0.023% (wt/wt) (2.3 g/10 kg chow) and 0.035% (wt/wt) (3.5 g/10 kg chow), respectively. At 8 weeks of age, rats in the Ang II group, Ang II + low-dose luteolin group, and Ang II + high-dose luteolin group were treated with Ang II ([Val^5^]-angiotensin II; WAKO Pure Chemical Industries, Ltd., Osaka, Japan) in phosphate buffered saline (PBS) (-) for 7 days administered via a subcutaneously-implanted Alzet osmotic mini-pump (model 2001, DURECT Corp. Cupertino, CA, USA). The Ang II infusion rate averaged 0.7 μg/kg/min [14]. The control group rats were treated with PBS (-) for 7 days via a subcutaneously-implanted osmotic mini-pump. The diet was not changed in any group after implantation. Tail blood pressure and pulse rate were measured in conscious rats noninvasively using an Indirect Blood Pressure Meter (Softron, Tokyo, Japan). Echocardiographic assessment was performed under anesthesia (400 mg/kg of chloral hydrate I.P.). Standard parasternal short-axis images were recorded using a Sonos 5500 ultrasound system (Philips Medical Systems, Amsterdam, The Netherlands) with a 15 MHz sector probe. The short-axis image at the mid-papillary muscle level was used for M-mode assessment. Echocardiographic measurements were averaged from at least 3 separate cardiac cycles. After the echocardiographic assessment, the rats were over-anesthetized and the hearts were carefully excised and weighed.

### Evaluation of oxidative stress in the cardiac ventricle

Dihydroethidium, an oxidative fluorescent dye, was used to evaluate O_2_^-^ *in situ* generation in the left ventricular tissue. Briefly, unfixed fresh tissue embedded in optimal cutting temperature (OCT) compound was frozen and cut into 30-μm-thick slices and mounted on slides. Each slice was submerged in 2 μmol/L of dihydroethidium (Life Technologies Corp., Carlsbad, CA, USA) in modified HEPES buffer and incubated at 37°C for 20 min. Oxidative fluorescence intensity was detected with a fluorescence microscope (Keyence Biozero, Keyence Co., Osaka, Japan) using a 525–540 nm band-pass excitation filter. Images were stored digitally. Fluorescence intensity was quantified microscopically from at least 3 random microscopic fields per tissue section using a computerized image analysis system (Image J 1.31, National Institutes of Health, USA).

### Pathological assessment of cardiac ventricle

Sections of rat ventricles were preserved in 20% formaldehyde solution and embedded in paraffin. Cross sections (6 μm) were cut and stained with Masson-trichrome staining. The area of stained fibrotic myocardium in the left ventricle was quantified in each image (40×magnification, 16-bit, RGB) split into blue-channel with a threshold of 120–180 using a computerized image analysis system (Image J 1.31) and the percent fibrosis area was calculated by dividing fibrotic pixels by total tissue pixels. In the cross sections stained with hematoxylin-eosin staining, the diameter of cardiomyocytes in the left ventricle was evaluated.

### Quantitative RT-PCR of ventricular tissue

The rat ventricles were rapidly frozen in RNAlater (Life Technologies Corp.) and homogenized using a MM-300 glass homogenizer (QIAGEN K.K., Tokyo, Japan) and then the total RNA was purified using a commercially available RNeasy Fibrous Tissue Midi Kit (QIAGEN K.K.). Reverse transcription of 1 μg RNA to cDNA was performed using SuperScript III First-Strand Synthesis SuperMix (Life Technologies Corp.) and Gene Amp PCR System 9700 (Life Technologies Corp.). For quantitation of the transcripts, real-time PCR analysis was performed using FastStart Universal SYBR Green Master (ROX) (Roche Diagnostic K.K., Tokyo, Japan) in a 7500 Real Time PCR System with Sequence Detection Software Ver.2.0 (Life Technologies Corp.). The expression level of each gene was normalized to that of glyceraldehyde-3-phosphate dehydrogenase (GAPDH). The sequences of PCR primers for transforming growth factor-β1 (TGFβ1), connective tissue growth factor (CTGF), collagen 1, collagen 3, tumor necrosis factor-α (TNFα), monocyte chemotactic protein-1 (MCP1), NADPH oxidase subunit 2 (Nox2), NADPH oxidase subunit 4 (Nox4), atrial natriuretic peptide (ANP), brain natriuretic peptide (BNP), and GAPDH are shown in the S1 Table.

### Measurement of plasma luteolin concentrations

Sample preparation was performed according to a method described previously [15]. Briefly, 110 μL of 75 mmol/L phosphate buffer (pH 6.8) containing 50 U β–glucuronidase was transferred to a 1 mL glass tube and then a 20 μL aliquot of rat plasma sample was added. The resulting solutions were incubated to hydrolyze the luteolin conjugates at 37°C for 1 hr. A 500 μL volume of ethyl acetate as an extraction solvent was added after 130 μL of 10 mmol/L oxalic acid and 100 μL of methanol or standard solution were added. The sample was vortexed, followed by ultrasonic vibrations for 15 min and then centrifugation at 10,000 *g* for 5 min. The organic layer (approximately 400 μL) was transferred to a 1 mL glass tube and evaporated to dryness using a centrifuge concentrator. The dried extract was reconstituted in 100 μL of methanol and then centrifuged at 10,000 *g* for 5 min. A 50 μL aliquot of supernatant of reconstituted sample solution was injected into a chromatographic system.

The HPLC-MS/MS system consisted of a Prominence micro-LC system (Shimadzu, Kyoto, Japan) and an API 3200 tandem mass spectrometer (Applied Biosystems, CA, USA) with (-) electrospray ionization (ESI). The analytical method was as follows; column: C-18 column-Atlantis T3 (2.1*150 mm, 3 μm) (Waters, Milford, MA, USA), mobile phase: (A) 0.1% formic acid/ H_2_O and (B) 0.1% formic acid/acetonitrile, gradient (in buffer A): 0–2 min, 50–95% B; 2–5 min, 95% B; 5–5.01 min, 95–50% B; 5.01–15 min, 50% B, flow rate: 0.2 mL/min, temperature: 40°C, injection volume: 5 μL. The mass spectrometer was operated under MRM mode with a collision energy of -44 eV (negative mode). The transitions (precursor to product) monitored were *m*/*z* 284.71→132.9. Chromatograms were integrated using ANALYST version 1.5 software (AB SCIEX, Framingham, MA, USA). Stock solution of luteolin in methanol (1,000 ng/mL) was diluted with methanol to prepare the following standard solutions: 0.8, 3.1, 12.5, 50.0, and 250.0 ng/mL.

### Cell Culture

Adult rat cardiac fibroblasts purchased from Cell Applications, Inc. (San Diego, CA, USA) were cultured in the manufacturer recommended medium (CAR116K500) to sub-confluence at 37°C in a humidified atmosphere of 5% CO_2_. Cells from passages 6–10 were used for experiments. Cells were starved in the recommended medium (CAR115500) for 24 hr before administration of H_2_O_2_ or vehicle. Cells were pretreated with the indicated doses of luteolin (Sigma-Aldrich Co. LLC) for 24 hr and then exposed to H_2_O_2_ or vehicle.

### Quantitative RT-PCR of cultured cardiac fibroblasts

Cellular total RNA was purified using an RNeasy Mini Kit (QIAGEN K.K.). Reverse transcription and real-time PCR analysis were performed as described above. The expression level of each gene was normalized to that of β-actin. The sequences of PCR primers for TGFβ1, CTGF, collagen 1, collagen 3, Nox2, Nox4, and β-actin are shown in the S1 Table.

### Western Blotting Analysis

Cells were washed with ice-cold PBS and lysed using a buffer composed of 25 mmol/L Tris (pH 7.4), 10 mmol/L EDTA, 10 mmol/L EGTA, 1 mmol/L sodium orthovanadate, 10 mmol/L sodium pyrophosphate, 100 mmol/L sodium fluoride, 1 mmol/L phenylmethylsulfonyl fluoride, and 1% Triton X-100, and supplemented with a complete protease inhibitor mixture (Roche Diagnostic K.K.) and placed on ice for 10 min. After centrifugation at 15,000 *g* for 10 min at 4°C, the supernatant was clarified and the protein concentration was determined with a BCA kit. After being normalized with total protein content, samples were added to 5-fold Laemmli buffer (200 mmol/L Tris-HCl, pH 6.8, 10%SDS, 50% glycerol, bromophenol blue with 10% 2-mercaptoethanol) and boiled for 5 min. Samples were subjected to SDS-PAGE on 10% gels which were blotted onto nitrocellulose for Western blotting. Membranes were blocked by incubation for 1 hr in TBST with 5% BSA and were then incubated for 1 hr at room temperature or overnight at 4°C with Phospho-ERK1/2 antibody, ERK1/2 antibody, Phospho-JNK antibody, JNK antibody, Phospho-p38MAPK antibody, or p38MAPK antibody (Cell Signaling Technology, Inc., Danvers, MA, USA). After washing twice in TBST, the membranes were incubated for 1 hr at room temperature with horseradish peroxidase-conjugated secondary antibody. Membrane-bound secondary antibodies were visualized using an ECL chemiluminescence kit (GE Healthcare Japan Corp., Tokyo, Japan).

### Statistical analysis

All data are presented as the mean ± SD. Differences among groups were evaluated by one-way ANOVA followed by Scheffe’s test for multiple comparisons using SPSS (Ver.19.0, SPSS Inc., Chicago, IL, USA). All p values were two-sided and considered statistically significant at less than 0.05.

## Results

### Plasma luteolin concentrations, blood pressure, and echocardiographic findings

The Concentration of plasma luteolin in the rats fed the 0.023% luteolin diet and those fed the 0.035% luteolin diet for 1 week was 217.2 ± 71.3, and 324.2 ± 41.0 ng/mL, respectively (0.023% luteolin diet (n = 5) versus 0.035% luteolin diet (n = 5); P = 0.020). Plasma luteolin levels in rats not fed luteolin diet were below the detection threshold of 0.3 ng/mL.

**Fig 1:**
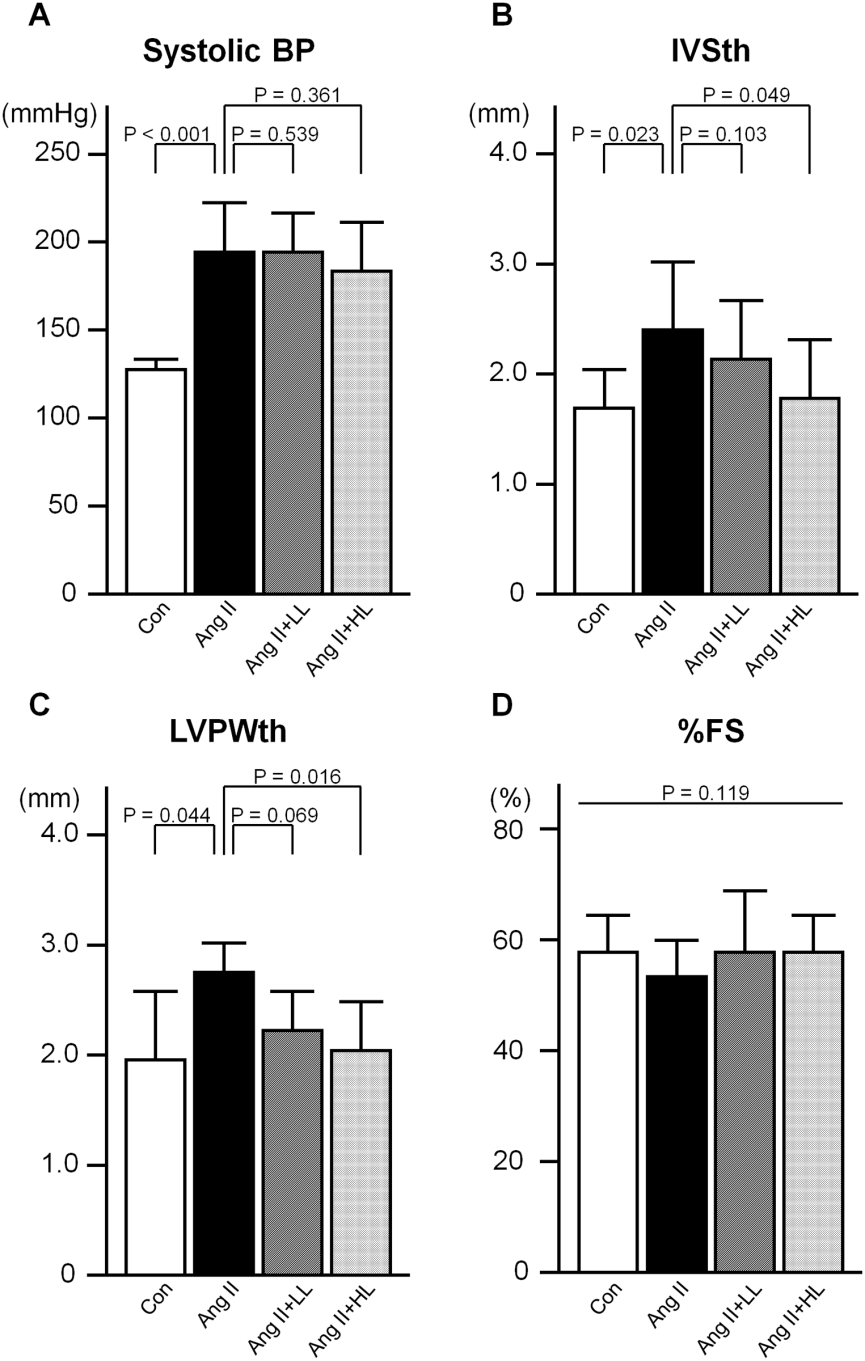
Systolic blood pressure and echocardiographic findings. A, Systolic blood pressure. B, Interventricular septal thickness. C, Left ventricular posterior wall thickness. D, Fractional shortening percentage. Con; the control group, Ang II; the Ang II group, Ang II+LL; the Ang II with pre-administration of lower-dose (0.023%) luteolin diet group, Ang II+HL; the Ang II with pre-administration of higher-dose (0.035%) luteolin diet group. Values are presented as mean ± SD. n = 6–11 per group.

Systolic blood pressure levels were significantly elevated by Ang II infusion. Oral pre-administration of luteolin did not lower the high blood pressure induced by Ang II (Fig 1A). There was no difference in pulse rate among the groups *(data not shown)*. Echocardiographic analysis revealed left ventricular wall thickness was significantly greater in the Ang II group than in the control group. The augmented wall thickness observed in the Ang II group was significantly ameliorated by oral pre-administration of the 0.035% luteolin diet, but not by that of the 0.023% luteolin diet (Fig 1B and 1C). There was no difference in fractional shortening percentage (Fig 1D) and end-diastolic left ventricular diameter *(data not shown)* among the groups.

### Pathological findings

**Fig 2:**
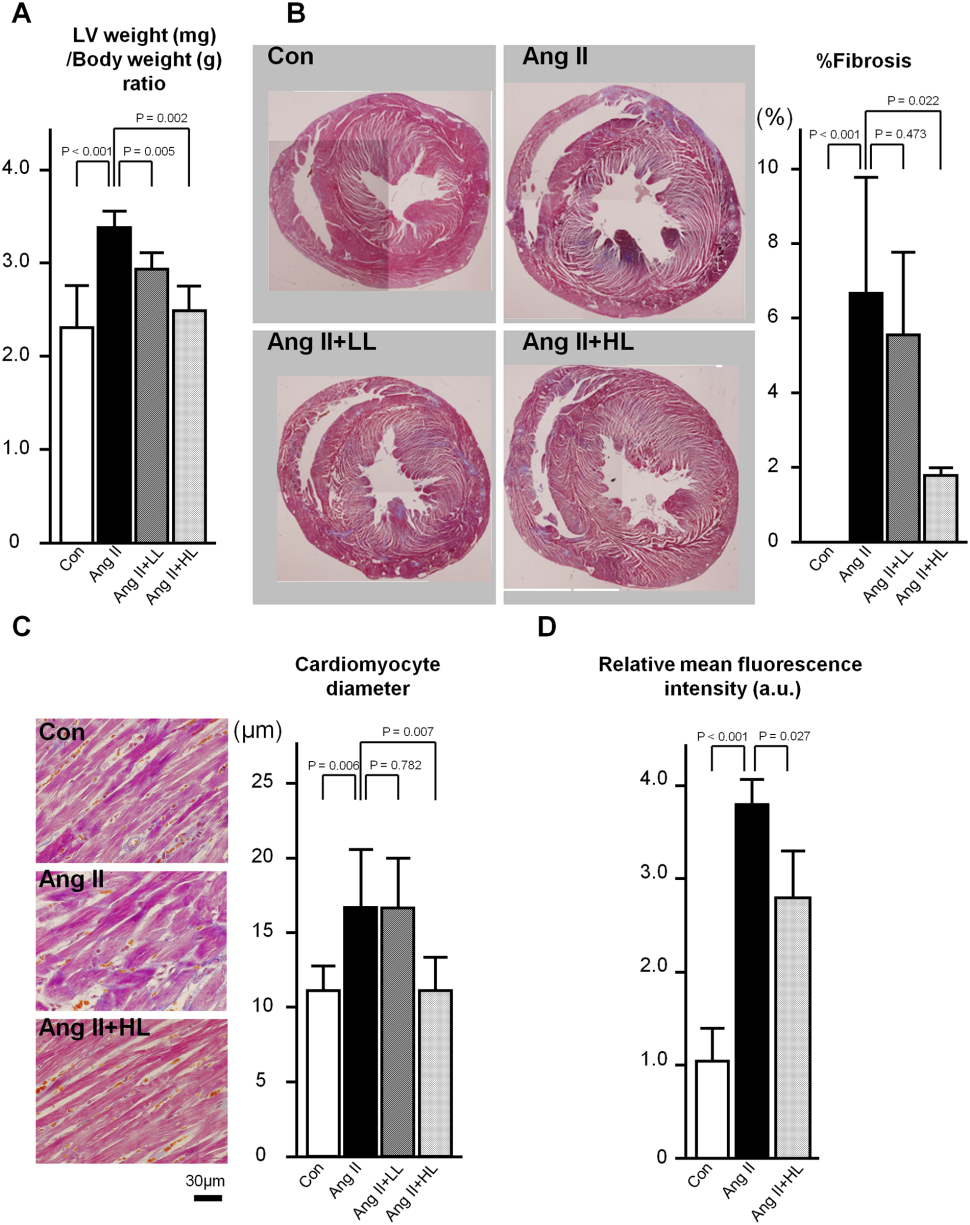
Pathological findings and evaluation of oxidative state. A, Left ventricular weight (mg)/body weight (g) ratio. B, Left, Representative mid-ventricular cross sections in Masson-trichrome staining. Right, Percent fibrosis of left ventricles. C, Left, Representative mid-ventricular cross sections in hematoxylin-eosin staining. Right, Cardiomyocyte diameter in left ventricles. D, Relative mean oxidative fluorescence intensity in left ventricles. Mean of the results in the control group was assigned an arbitrary value of 1.0. Abbreviations for all groups are the same as those in Fig 1. Values are presented as the mean ± SD. n = 6–11 (A-C) and 3–5 (D) per group.

The left ventricular weight was greater in the Ang II group than in the control group (P < 0.001). The increased left ventricular weight observed in the Ang II group was significantly ameliorated by oral pre-administration of the luteolin diet (P = 0.005 for 0.023% luteolin diet group, and P = 0.002 for 0.035% luteolin diet group) (Fig 2A). The results of histopathological analysis showed the fibrosis of the left ventricular wall was significantly exacerbated in the Ang II group, which was significantly restored by oral pre-administration of the 0.035% luteolin diet, but not by that of the 0.023% luteolin diet (Fig 2B). Cardiomyocyte diameter was significantly increased in the Ang II group compared with the control group, and was significantly restored by oral pre-administration of the 0.035% luteolin diet, but not by the 0.023% luteolin diet (Fig 2C).

### Oxidative state

The oxidative fluorescence intensity was significantly greater in the ventricular walls of the Ang II group rats than in those of the control group rats (P < 0.001). The augmented oxidative state observed in the Ang II group was significantly ameliorated by oral pre-administration of the 0.035% luteolin diet (P = 0.027) (Fig 2D).

### Gene expression levels in rat cardiac ventricles

**Fig 3:**
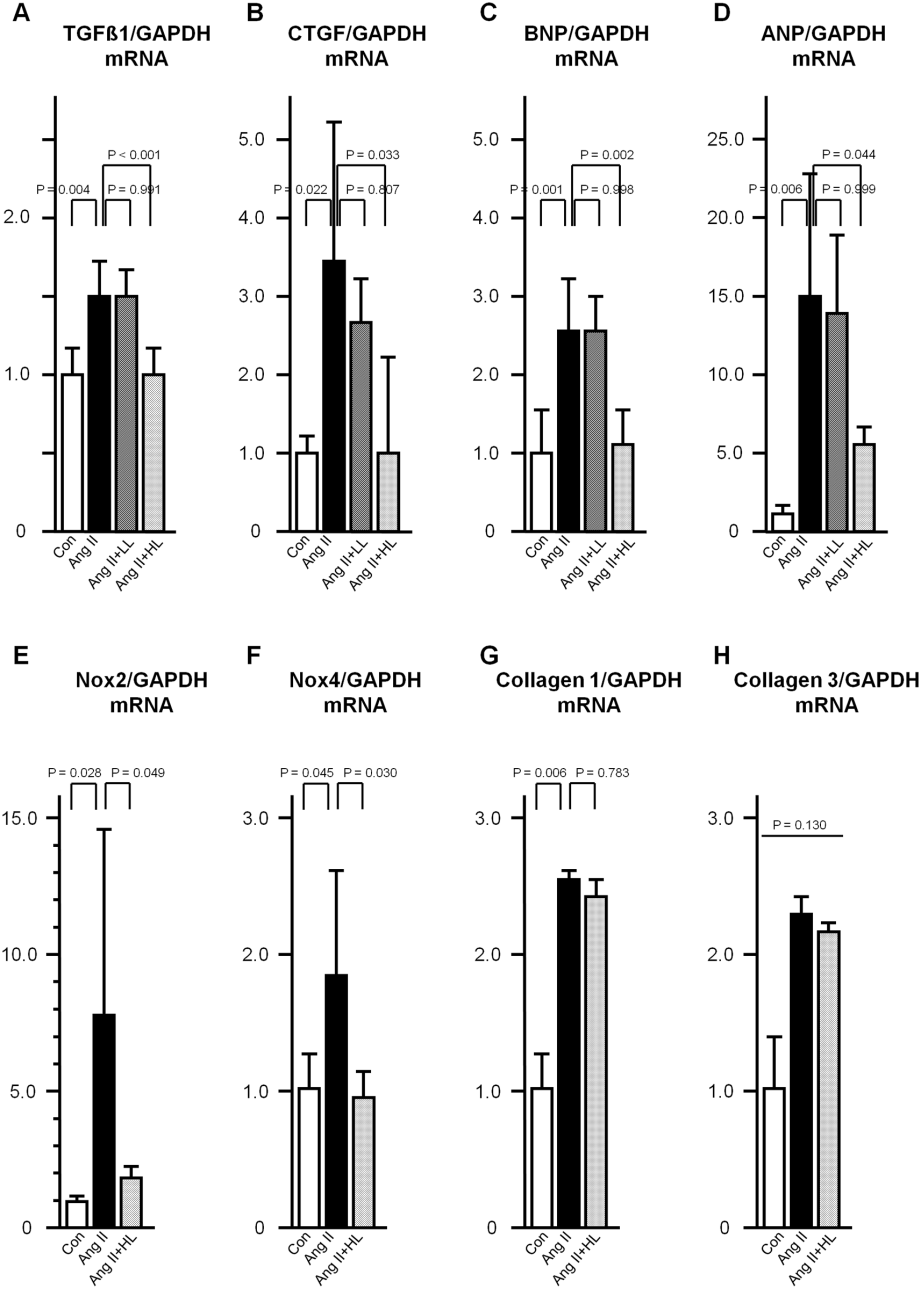
Gene expression levels in rat cardiac ventricles. A, TGFβ1. B, CTGF. C, BNP. D, ANP. E, Nox2. F, Nox4. G, collagen 1. H, collagen 3. Gene expression levels of each gene normalized to those of GAPDH in rat cardiac ventricles are presented as fold-changes. Abbreviations for all groups are the same as those in Fig 1. Values are presented as the mean ± SD. n = 6–9 per group.

Gene expression levels of TGFβ1, CTGF, collagen 1, collagen 3, TNFα, MCP1, Nox2, Nox4, ANP, and BNP in rat cardiac ventricles were evaluated. Expression levels of TGFβ1, CTGF, collagen 1, Nox2, Nox4, ANP, and BNP were significantly augmented in the Ang II group as compared with the control group. Oral pre-administration of the 0.035% luteolin diet significantly abrogated the Ang II-induced increased expression levels of TGFβ1, CTGF, Nox2, Nox4, ANP, and BNP (Fig 3). There were no significant differences in the expression levels of collagen 3, TNFα, and MCP1 among the groups (Fig 3 and S1 Fig).

### Experimental findings in cultured rat cardiac fibroblasts

**Fig 4:**
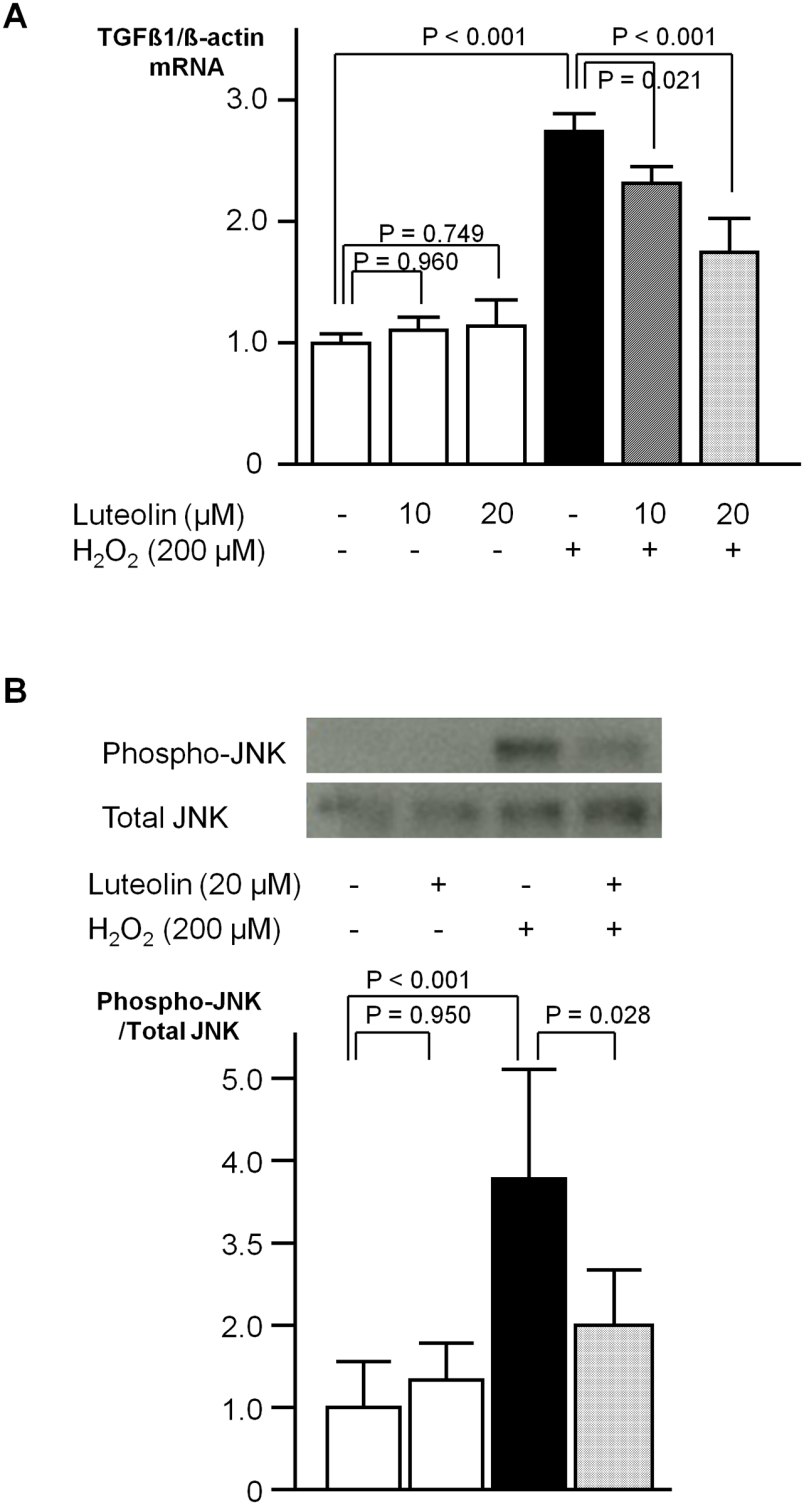
Experimental findings in cultured rat cardiac fibroblasts. A, Gene expression levels of TGFβ1 normalized to those of β-actin at 24 hr after H_2_O_2_ treatment are presented as fold-changes. Values are presented as the mean ± SD. n = 6–9 per group. B, Upper, Representative photographs of Phospho-JNK and total JNK at 15 min after H_2_O_2_ treatment. Lower, Expression levels of Phospho-JNK divided by those of total JNK at 15 min after H_2_O_2_ treatment are presented as fold-changes. Values are presented as the mean ± SD. n = 6 per group.

In cultured rat cardiac fibroblasts, the gene expression levels of TGFβ1 were significantly increased by treatment with H_2_O_2_ (200 μmol/L), while those of CTGF, collagen 1, collagen 3, Nox2, and Nox4 were not. At 24 hr after the administration of H_2_O_2_, the TGFβ1/β-actin ratio was 2.7-fold higher than the baseline ratio, and could be restored by pretreatment with 10 or 20 μmol/L of luteolin in a dose-dependent manner (Fig 4A). Treatment with H_2_O_2_ (200 μmol/L) induced the phosphorylation of ERK, JNK, and p38MAPK. Phosphorylation of JNK was significantly restored by pretreatment with 20 μmol/L of luteolin (Fig 4B), while the phosphorylation of ERK1/2 or p38MAPK was not (S2 Fig). At 15 min after the administration of H_2_O_2_ (200 μmol/L), the phosphorylated JNK/total JNK ratio was 3.7-fold higher than the baseline ratio, and could be significantly restored by pretreatment with 20 μmol/L of luteolin.

## Discussion

The findings of the present study demonstrate that the oral administration of luteolin has protective actions against Ang II-induced cardiac remodeling, which could be mediated through attenuation of oxidative stress.

Cardiac fibroblasts are the most prevalent cell type in the myocardium [16,17] and play a central role in the maintenance of the extracellular matrix in the normal heart. Together with cardiomyocytes, they act as the key determinants of cardiac development, myocardial structure, cell signaling, and electromechanical function [16,18]. Under some pathological conditions, cardiac fibroblasts can proliferate and increase the deposition of extracellular matrix proteins, leading to interstitial fibrosis, which enhances intrinsic myocardial stiffness and results in diastolic dysfunction, accounting for 30% to 50% of congestive heart failure in a clinical setting [19].

Systemically and locally, activation of the renin-angiotensin system plays an important pathophysiological role in cardiovascular diseases, and blockade of Ang II, a key profibrotic activator of cardiac fibroblasts, has been widely shown to regress cardiac remodeling [20]. Ang II is known to stimulate NADPH oxidase, which generates ROS in cardiac fibroblasts [7]. Activation of NADPH oxidase by Ang II plays a crucial role in the development of cardiovascular diseases [6,21,22]. ROS can act as second messengers that regulate the various intracellular signal transduction cascades and the activity of various transcription factors. One of the potential targets involved in these redox-sensitive mechanisms in fibroblast activation appears to be the activation of mitogen-activated protein kinase (MAPK) pathways [7,8,23]. Also, Ang II-induced fibroblast activation includes the production and release of a potentially profibrotic cytokine TGFβ1 by cardiac fibroblasts [24,25]. Actually, functional blockade of TGFβ could attenuate cardiac remodeling and cardiac dysfunction [26,27]. Blockade of the Ang II-TGFβ1 signaling pathway has consistently been demonstrated to be effective for the prevention of cardiac fibrosis in experimental animals [28–30]. In addition, even the cancellation of oxidative stress, which is thought to play a pivotal role in the Ang II-TGFβ1 signaling pathway, could reverse the cardiac fibrosis and prevent heart failure [21,31–34]. The results of the present study clearly demonstrate that the food-derived flavonoid luteolin functions as an antioxidant and restores the Ang II-induced increase in gene expression of TGFβ1 and CTGF [35], thereby protecting against cardiac fibrosis and hypertrophy. Resveratrol and curcumin have been shown to prevent cardiac remodeling in other animal models [36–39], however, to the best of our knowledge, no previous study has found a food-derived compound to be effective in an animal model with Ang II. As Ang II is a key regulator of the cardiovascular system [40], our findings on the Ang II-infused model are expected to contribute to the prevention and treatment of a variety of cardiovascular disorders.

The antifibrotic effects of luteolin on the myocardium was clearly demonstrated here, which are consistent with the antifibrotic effect on the other organs [12,13]. Why were the gene expression levels of collagen 1 and 3 in rat cardiac ventricules not lowered in the 0.035% luteolin diet group with less fibrotic cardiac tissue? This might be the situation in hearts with late disease as opposed to earlier findings of regulation of mRNA levels of collagens. Further analyses in earlier phases are warranted.

**Fig 5:**
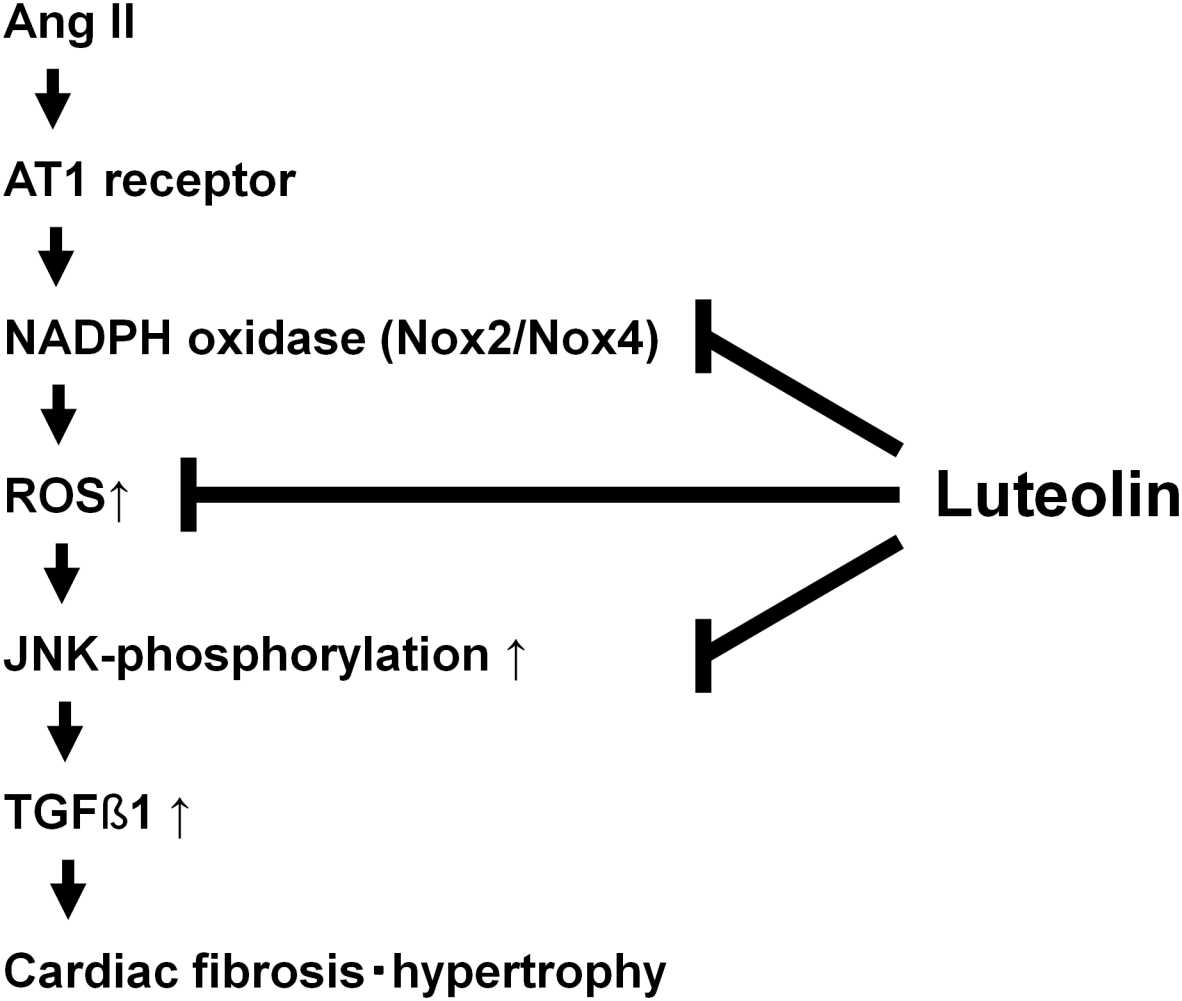
Schematic diagram of cardioprotective action of luteolin against Ang II excess.

In our experiments using cultured cardiac fibroblasts, the induction of TGFβ1 by administration of Ang II (~10 μM) was not necessarily reproduced (*data not shown*). In the whole hearts, regulation of the Ang II-TGFβ1 pathway in cardiomyocytes influences the extent of cardiac fibrosis in a paracrine manner. Also, macrophages and platelets could be the sources of TGFβ1. Due to the lack of these paracrine effects, in the culture of cardiac fibroblasts alone without other cells, it was not possible to reproduce the increase in gene expression of TGFβ1 by the administration of Ang II in a stable or consistent manner. Indeed, a suppressive effect of luteolin on the Ang II-induced hypertrophic growth of cardiomyocytes was observed in this model (Fig 2C), implying that our *in vivo* data should be interpreted at least in terms of a relationship between cardiac fibroblasts and cardiomyocytes. Finally, instead of Ang II, a well-known oxidant H_2_O_2_ was administered here to demonstrate the suppression of redox-dependent TGFβ1 induction by the pretreatment with luteolin. According to our *in vitro* data, this suppressive effect of luteolin is thought to be related to its suppression of H_2_O_2_-induced JNK phosphorylation, consistent with the previous findings that luteolin suppresses the adipocyte-dependent activation of macrophages through the inhibited JNK phosphorylation [41]. Taken together, the oxidative properties of Ang II play a pivotal role in cardiac fibrosis and hypertrophy, which might be effectively restored by luteolin through the suppression of JNK phosphorylation and TGFβ1 induction. Also, in this study, the gene expression levels of NADPH oxidase subunits augmented by Ang II were restored by pretreatment with luteolin, suggesting that the regulation of NADPH oxidase might be relevant to the antioxidative effect of luteolin (Fig 5).

Our results indicate that oral pretreatment with luteolin is expected to become a novel and valuable therapeutic approach to suppress cardiac remodeling (e.g. human hypertensive heart disease). In a recent report [42], luteolin was also shown to protect the myocardium against ischemia-reperfusion injury through its antioxidant properties. Cardiovascular protection is required throughout the entire life and this could be most easily and safely accomplished by identifying and administrating protective agents of low toxicity that are already present in our diet. One of the major advantages of luteolin is related to the fact that it is already a component of the human daily diet and is therefore expected to be relatively safe for chronic administration. Our present study results might partly explain the mechanistic rationale for the recommendation that vegetable intake is useful for risk reduction in cardiovascular disease [43]. However, to achieve the cardioprotective effects observed here, much higher quantities than those in daily vegetable intake might be required. Notably, in our analysis, the protective effects of the 0.023% luteolin diet corresponding to lower levels of plasma luteolin were insufficient by contrast with those of the 0.035% luteolin diet, suggesting that its effects can be exerted in a dose-response fashion. Considering the unique metabolic rates in different species [44], based on our present study, we cannot speculate the quantity to be orally administered in humans. Further studies are warranted to estimate the quantity of luteolin supplementation required to exert a cardioprotective effect in humans.

## Conclusions

Oral supplementation of luteolin (3’,4’,5,7-tetrahydroxyflavone) was found to protect against cardiac fibrosis and hypertrophy in an Ang II-infused rat model, which could be mediated through attenuation of oxidative stress.

## Supporting Information

S1 ARRIVE Checklist The ARRIVE Guidelines Checklist. (PDF)

S1 Fig Gene expression levels of TNFα and MCP1 in rat cardiac ventricles. Gene expression levels of each gene normalized to those of GAPDH in rat cardiac ventricles are presented as fold-changes. Abbreviations for all groups are the same as those in Fig 1. Values are presented as the mean ± SD. n = 6–9 per group.
(TIF)

S2 Fig Phosphorylation of ERK1/2 and p38MAPK in cultured rat cardiac fibroblasts. Expression levels of Phospho-ERK1/2 and Phospho-p38MAPK at 15 min after H_2_O_2_ treatment are shown after being divided by those of total ERK1/2 and total p38MAPK, respectively. Values are presented as the mean ± SD. n = 6 per group.
(TIF)

S1 Table Primer sequences used for quantitative RT-PCR. (PDF)
