# Supplementary figures and images for: A Food-Derived Flavonoid Luteolin Protects against Angiotensin II-Induced Cardiac Remodeling
Source: PLoS One. 2015 Sep 1;10(9):e0137106. doi: 10.1371/journal.pone.0137106 (PMC4556625; doi:10.1371/journal.pone.0137106)

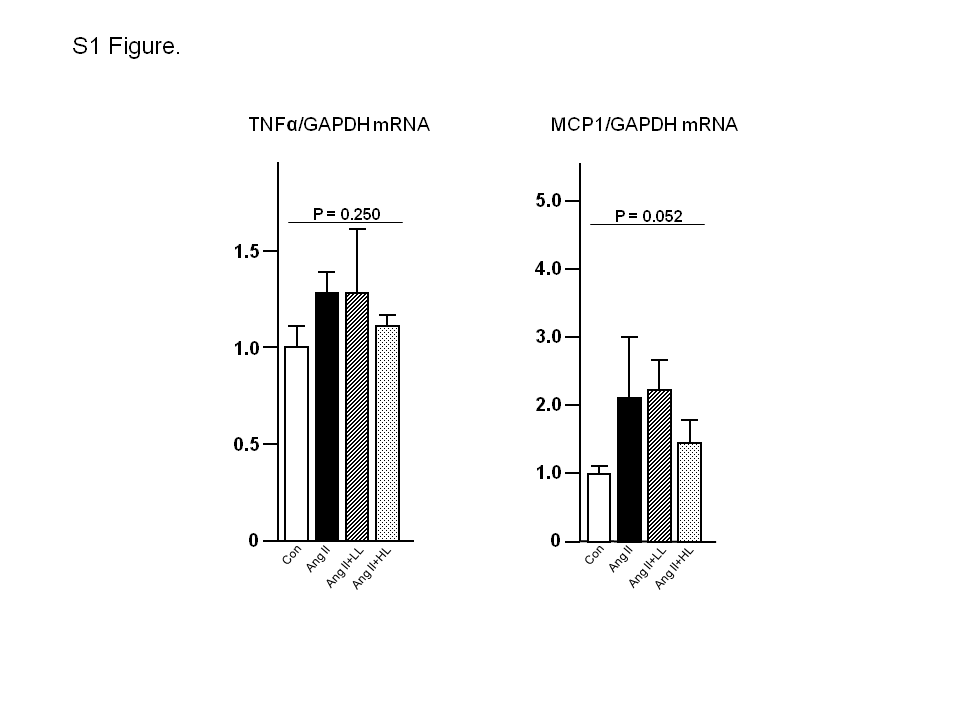

Supplement: S1 Fig — Gene expression levels of each gene normalized to those of GAPDH in rat cardiac ventricles are presented as fold-changes. Abbreviations for all groups are the same as those in Fig 1. Values are presented as the mean ± SD. n = 6–9 per group. (TIF) [file pone.0137106.s002.tif]

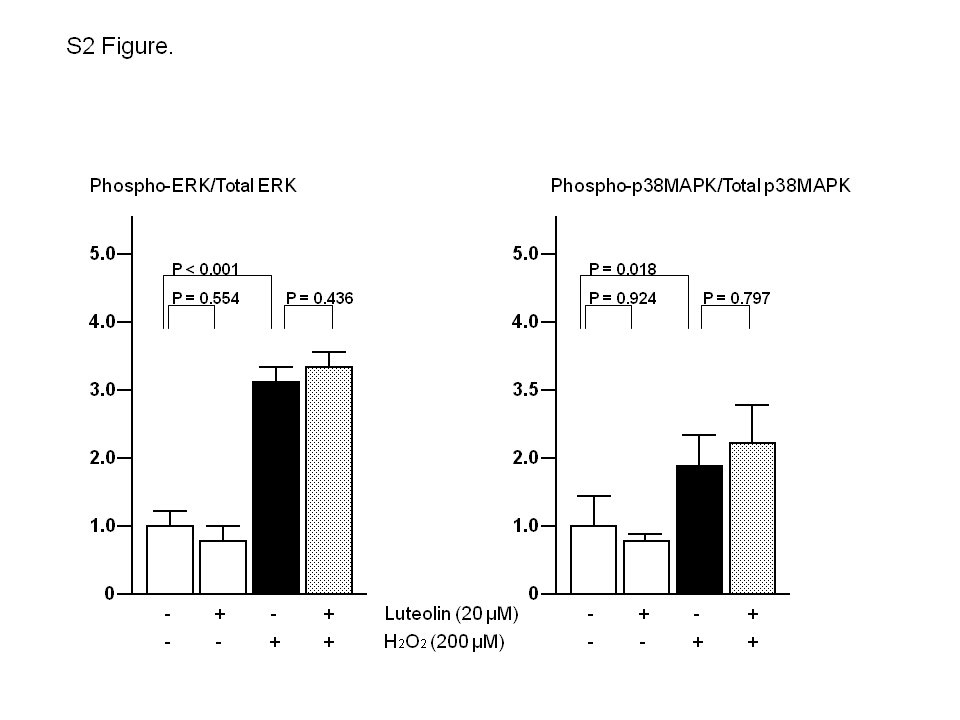

Supplement: S2 Fig — Expression levels of Phospho-ERK1/2 and Phospho-p38MAPK at 15 min after H2O2 treatment are shown after being divided by those of total ERK1/2 and total p38MAPK, respectively. Values are presented as the mean ± SD. n = 6 per group. (TIF) [file pone.0137106.s003.tif]
