# Supplementary material for: A Food-Derived Flavonoid Luteolin Protects against Angiotensin II-Induced Cardiac Remodeling
Source: PLoS One. 2015 Sep 1;10(9):e0137106. doi: 10.1371/journal.pone.0137106 (PMC4556625; doi:10.1371/journal.pone.0137106)
Supplement: S1 Table — (PDF) [file pone.0137106.s004.pdf]

S1 Table. Primer sequences used for quantitative RT-PCR

**Gene**	**Forward primer (5'-3')**	**Reverse primer (5'-3')**
rat TGFβ1 (*Tgfb1*)	ACCTGCAAGACCATCGACATG	CGAGCCTTAGTTTGGACAGGAT
rat CTGF (*Ctgf*)	AAGACCTGTGGGATGGGC	TGGTGCAGCCAGAAAGCTC
rat Collagen 1 (*Col1a1*)	CAACCTCAAGAAGTCCCTGC	AGGTGAATCGACTGTTGCCT
rat Collagen 3 (*Col3a1*)	CACCCCTCTCTTATTTTGGCAC	AGACTCATAGGACTGACCAAGGTAGTT
rat TNFα (*Tnf*)	CCCAGACCCTCACACTCAGAT	TTGTCCCTTGAAGAGAACCTG
rat MCP1 (*Ccl2*)	ATGCAGTTAATGCCCCACTC	TTCCTTATTGGGGTCAGCAC
rat Nox2 (*Cybb*)	ACTTCTTGGGTCAGCACTGG	GTTCCTGTCCAGTTGTCTTCG
rat Nox4 (*Nox4*)	TAGCTGCCCACTTGGTGAACG	TGTAACCATGAGGAACAATACCACC
rat ANP (*Nppa*)	ATCACCAAGGGCTTCTTCCT	TGTTGGACACCGCACTGTAT
rat BNP *(Nppb*)	ACAATCCACGATGCAGAAGCT	GGGCCTTGGTCCTTTGAGA
rat GAPDH (*Gapdh*)	ATGATTCTACCCACGGCAAG	CTGGAAGATGGTGATGGGTT
rat β-actin (*Actb*)	GGGAAATCGTGCGTGACATT	GCGGCAGTGGCCATCTC
